# Thermoelectric Transport Properties of Cu_4_Bi_4_Se_9_ Prepared by Mechanical Alloying and Hot Pressing

**DOI:** 10.3390/mi17050615

**Published:** 2026-05-17

**Authors:** Gyuseong Chu, Il-Ho Kim

**Affiliations:** Department of Materials Science and Engineering, College of Engineering, Korea National University of Transportation, Chungju 27469, Republic of Korea; a41780998@gmail.com

**Keywords:** thermoelectric, Cu_4_Bi_4_Se_9_, mechanical alloying, hot pressing

## Abstract

Single-phase Cu_4_Bi_4_Se_9_ was successfully synthesized through a simple and rapid process combining mechanical alloying (MA) and hot pressing (HP). The phase formation behavior, microstructural evolution, charge transport characteristics, and thermoelectric properties were systematically investigated. X-ray diffraction analysis as a function of MA time confirmed that all powders crystallized into a single orthorhombic phase with space group Pnma. No decompositions or secondary phases were observed after HP sintering, indicating high phase stability. Thermogravimetric and differential scanning calorimetric analyses revealed distinct endothermic peaks at 714–717 K for all samples, corresponding to the onset of the decomposition of Cu_4_Bi_4_Se_9_. Microstructural observations showed that the relative density decreased with increasing HP temperature (>573 K), accompanied by grain growth and pore formation, reflecting the competition between Cu–Se interdiffusion and pore coarsening during high-temperature sintering. Hall effect measurements indicated p-type conduction for all samples, with carrier concentrations on the order of 10^17^ cm^−3^ and carrier mobilities of approximately 10^2^ cm^2^ V^−1^ s^−1^. With increasing temperature, the electrical conductivity increased monotonically, while the Seebeck coefficient gradually decreased, resulting in a maximum power factor of 0.12 mW m^−1^ K^−2^ at 573 K. The total thermal conductivity remained extremely low, ranging from 0.33 to 0.48 W m^−1^ K^−1^, with the electronic contribution accounting for less than 10%, indicating that lattice thermal transport is dominant. The suppressed lattice thermal conductivity is attributed to the combined effects of Cu atomic rattling, asymmetric bonding induced by Bi 6s^2^ lone-pair electrons, and strong anharmonic phonon scattering arising from the complex crystal structure. Consequently, Cu_4_Bi_4_Se_9_ achieved a peak dimensionless figure of merit ZT of 0.19 in the temperature range of 573–623 K, demonstrating that the MA–HP process enables stable phase formation and competitive thermoelectric performance without post-annealing.

## 1. Introduction

Cu–Bi–Se-based compounds are ternary chalcogenides characterized by mixed ionic–covalent Cu–Se and Bi–Se bonding, which gives rise to complex crystal frameworks and intrinsically strong phonon scattering. As a result, these materials generally exhibit low lattice thermal conductivity, making them attractive candidates for thermoelectric applications [1,2]. Representative compounds reported to date include CuBi_3_Se_5_, CuBiSe_2_, Cu_3_BiSe_3_, Cu_9_BiSe_6_, Cu_4_Bi_4_Se_9_, and Cu_3_BiSe_4_ [3,4,5,6], many of which belong to quasi-diamond or pavonite-type homologous series characterized by layered or modular structural motifs. Early studies mainly addressed optoelectronic properties, whereas recent work has emphasized their potential as topological insulators and thermoelectric materials because of their low lattice thermal conductivity and unconventional electronic structures [7,8,9]. Within this materials family, Cu_4_Bi_4_Se_9_ has attracted particular attention as a model system for intrinsically low lattice thermal conductivity because it exhibits both pronounced structural complexity and strong coupling between charge carriers and the lattice. It crystallizes in the orthorhombic Pnma space group and adopts a layered architecture consisting of alternating PbS-type modules and Bi–Cu complex layers, a structural characteristic that effectively enhances phonon scattering.

Single-crystal X-ray diffraction analysis by Makovický et al. [7] revealed that Cu_4_Bi_4_Se_9_ is isotypic with Cu_4_Bi_4_S_9_, with charge neutrality maintained by monovalent Cu^+^ cations and ${Se}_{2}^{2-}$ dimers. This bonding configuration generates an asymmetric local potential that stabilizes the electronic structure while suppressing lattice vibrations. Based on this structural framework, Jiang et al. [8] systematically examined the thermoelectric properties of Cu_4_Bi_4_Se_9_ and reported an ultralow lattice thermal conductivity (κ_L_ < 0.4 W m^−1^ K^−1^), which they attributed to structural complexity, soft Cu–Se bonding, rattling-like motion of Cu atoms, and strong anharmonicity associated with stereochemically active Bi 6s^2^ lone-pair electrons. More generally, Peccerillo and Durose [9] showed that in Cu–Sb/Bi–S/Se chalcogenides, asymmetric bonding induced by ns^2^ lone-pair cations (Sb^3+^, Bi^3+^) enhances lattice distortion and phonon scattering while maintaining reasonably good charge transport. Consistently, Hobbis et al. [10] reported low lattice thermal conductivities (κ_L_ < 0.8 W m^−1^ K^−1^) in both Cu_4_Bi_4_S_9_ and Cu_4_Bi_4_Se_9_, attributing this behavior to low Debye temperatures, negligible electronic heat transport, and large unit cells with complex bonding environments. In addition, Han et al. [11] investigated defect structures and ion migration in Cu_4_Bi_4_Se_9_, providing further insight into the relationships among intrinsic defects, charge transport, and thermal stability in Cu–Bi–Se-based compounds.

Despite these promising intrinsic properties, synthesizing phase-pure and dense bulk materials in the Cu–Bi–Se system remains challenging. Various processing routes, including melt processing [12,13], thin-film deposition [14,15], quantum dot synthesis [16], single crystal growth [17], and multistep solid-state reactions [8,10], have been applied to Cu–Bi–Se-based compounds. However, these methods typically require prolonged high-temperature treatments, which can introduce kinetic constraints, Se volatilization, and secondary phases such as Cu_2_Se. In the case of Cu_4_Bi_4_Se_9_, bulk synthesis is particularly demanding because of its narrow phase stability range and sensitivity to compositional deviation. In contrast, the structural analogue Cu_4_Bi_4_S_9_ has been more extensively studied, both in terms of its crystal structure [18] and alternative synthesis routes, including solvothermal methods [19]. For Cu_4_Bi_4_Se_9_, a rapid and reliable synthesis strategy capable of producing bulk specimens with controlled microstructure and high phase purity has not yet been established. Developing simplified and scalable processing routes for such materials is essential for their practical implementation. Against this background, the present study adopts a combined mechanical alloying and hot pressing (MA–HP) approach to rapidly synthesize bulk Cu_4_Bi_4_Se_9_. By systematically examining phase evolution, microstructural development, charge transport behavior, and thermoelectric performance as functions of processing conditions, this work aims to elucidate the underlying processing–structure–property relationships in Cu_4_Bi_4_Se_9_. In particular, the novelty of this study lies not in compositional modification but in revealing how phase stability, densification behavior, and transport properties are interrelated under a non-equilibrium MA–HP route. This analysis identifies a clear trade-off between microstructural evolution and thermoelectric performance, providing practical insight into optimizing complex chalcogenide thermoelectric materials through simplified and scalable processing.

## 2. Experimental Procedure

Cu_4_Bi_4_Se_9_ samples were synthesized by mechanical alloying (MA) using high-purity elemental powders as starting materials and subsequently consolidated into dense bulk specimens by hot pressing (HP). High-purity Cu (99.9%, <45 µm; Kojundo Chemical Lab., Saitama, Japan), Bi (99.999%, <75 µm; Alfa Aesar, Ward Hill, MA, USA), and Se (99.9%, <75 µm; Kojundo Chemical Lab., Saitama, Japan) powders were used as precursors. The powders were weighed according to the stoichiometric ratio (Cu:Bi:Se = 4:4:9) and mixed thoroughly. Approximately 20 g of the powder mixture was loaded into a 500 mL hardened steel vial together with stainless steel balls (5 mm in diameter, total mass 400 g), corresponding to a ball-to-powder weight ratio (BPR) of 20:1. MA was performed under a high-purity argon atmosphere (Ar, 99.999%) using a planetary ball mill (Pulverisette 5, Fritsch GmbH, Idar-Oberstein, Germany) operated at 350 rpm for 3–12 h to obtain homogeneous Cu_4_Bi_4_Se_9_ powders. The as-milled powders were then transferred into a graphite die with an inner diameter of 10 mm and consolidated by HP at temperatures ranging from 523 to 623 K under an applied pressure of 70 MPa for 2 h. After sintering, the bulk samples were slowly cooled to room temperature under vacuum. The sample nomenclature used in this study is defined as follows. The notation “xH” refers to the duration of mechanical alloying, while “yH” in the hot-pressed samples indicates the holding time during the hot-pressing process. For example, MA350R6H denotes a sample prepared by mechanical alloying at 350 rpm for 6 h, and HP523K2H represents a sample hot-pressed at 523 K for 2 h after mechanical alloying.

The crystalline phases and structural characteristics of the samples were examined by X-ray diffraction (XRD; D8 Advance, Bruker Corp., Billerica, MA, USA) using Cu Kα radiation. Diffraction patterns were recorded over a 2θ range of 10–90° with a step size of 0.02° and a counting time of 0.4 s per step. The XRD data were analyzed by Rietveld refinement using the TOPAS software (version 4.1, Bruker Corp., Billerica, MA, USA) to determine lattice parameters and phase purity. The relative density of the bulk samples was evaluated by comparing the measured density, calculated from the mass and geometric volume, with the theoretical density of Cu_4_Bi_4_Se_9_ (7.278 g cm^−3^) [7]. Crystallite sizes were estimated from the full width at half maximum (FWHM) of the diffraction peaks using Lorentzian peak-shape fitting. The microstructures were examined using scanning electron microscopy (SEM; Prisma E, Thermo Fisher Scientific Inc., Waltham, MA, USA).

The thermal stability and phase transition behavior of the samples were evaluated by simultaneous thermogravimetric analysis and differential scanning calorimetry (TG–DSC; TGA/DSC1, Mettler Toledo Inc., Columbus, OH, USA). Measurements were performed under a high-purity nitrogen atmosphere (N_2_, 99.999%) at a heating rate of 5 K min^−1^ up to 850 K. Charge transport properties were characterized by measuring the Hall coefficient (R_H_) using the van der Pauw method with a Hall effect system (Model 7065, Keithley Instruments Inc., Cleveland, OH, USA). Hall measurements were conducted at room temperature under a magnetic field of 1 T with repeated measurements (20 times for each sample), and the reported values represent the average with standard deviation. In addition, the measurement uncertainty was evaluated by considering contact resistance variation and sample geometry effects. The carrier concentration (n) and mobility (μ) were calculated using the relations n = 1/(e·R_H_) and μ = σ·R_H_, where e is the elementary charge and σ is the electrical conductivity. The Seebeck coefficient (α) and electrical conductivity were measured simultaneously over the temperature range of 323–623 K using a ZEM3 system (Advance-Riko Inc., Yokohama, Japan) under a helium atmosphere. For each temperature point, measurements were repeated at least five times, and only data satisfying instrument-defined stability criteria were accepted. The power factor (PF), which represents the electrical contribution to thermoelectric performance, was calculated according to PF = α^2^·σ. The thermal diffusivity (D) was measured using a laser flash analyzer (LFA717 HyperFlash, Netzsch Gerätebau GmbH, Selb, Germany) with a graphite coating applied to the sample surfaces to ensure accurate emissivity, and the specific heat capacity (c_p_) was estimated based on the Dulong–Petit approximation. The thermal conductivity (κ) was calculated using κ = d·c_p_·D, where d is the sample density. Finally, the dimensionless thermoelectric figure of merit (ZT) was determined over the temperature (T) range of 323–623 K using ZT = α^2^·σ·κ^−1^·T. All thermoelectric measurements were performed following prior calibration using certified standard reference materials provided by the manufacturer. At each temperature, measurements were automatically repeated at least five times, and only data satisfying the predefined stability criteria were accepted, ensuring reproducibility within instrumental uncertainty. Measurement uncertainties were evaluated by considering factors such as radiative heat loss, detector sensitivity, contact resistance, and sample geometry, and were estimated to be within ±5% for electrical properties and ±3% for thermal diffusivity.

## 3. Results and Discussion

**Figure 1:**
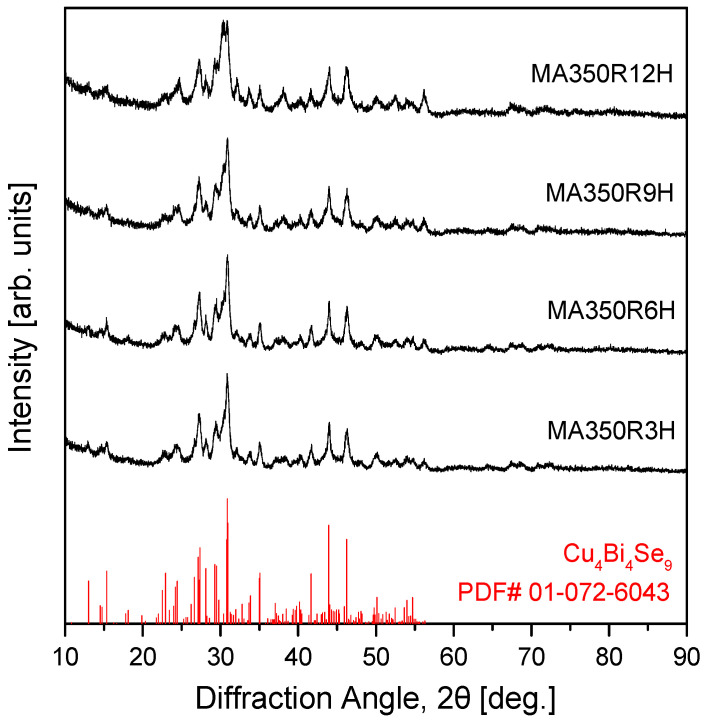
XRD patterns of Cu_4_Bi_4_Se_9_ powders mechanically alloyed for 3–12 h. All samples exhibited single-phase orthorhombic Cu_4_Bi_4_Se_9_ (space group Pnma, PDF# 01-072-6043) without detectable impurities.

Figure 1 shows the XRD patterns of the Cu_4_Bi_4_Se_9_ powder samples as a function of MA time (3–12 h). All samples exhibited diffraction peaks corresponding exclusively to single-phase Cu_4_Bi_4_Se_9_, in good agreement with the ICDD standard pattern (PDF# 01-072-6043), and all reflections could be indexed to the orthorhombic crystal system with space group Pnma, consistent with the structure reported by Makovický et al. [7]. In addition to confirming phase formation, the evolution of peak width and intensity with MA time provides insight into the microstructural effects induced by high-energy milling. At shorter milling times, peak broadening arises from reduced coherent domain size and increased microstrain caused by severe plastic deformation and high defect density. With prolonged milling, partial defect rearrangement and local stress relaxation may occur through repeated cold welding and fracture processes. The 6 h sample exhibited relatively sharp and well-defined reflections, indicating that phase formation is essentially complete under these conditions. The 9 and 12 h samples showed comparable peak sharpness and crystallinity without further phase evolution, suggesting that additional milling beyond 6 h offers limited structural improvement. This behavior indicates a saturation regime in which further mechanical energy input primarily maintains the existing defect structure rather than enhancing crystallinity. Accordingly, an MA time of at least 6 h can be considered a practical threshold for achieving a structurally stable Cu_4_Bi_4_Se_9_ phase under the present milling conditions.

**Figure 2:**
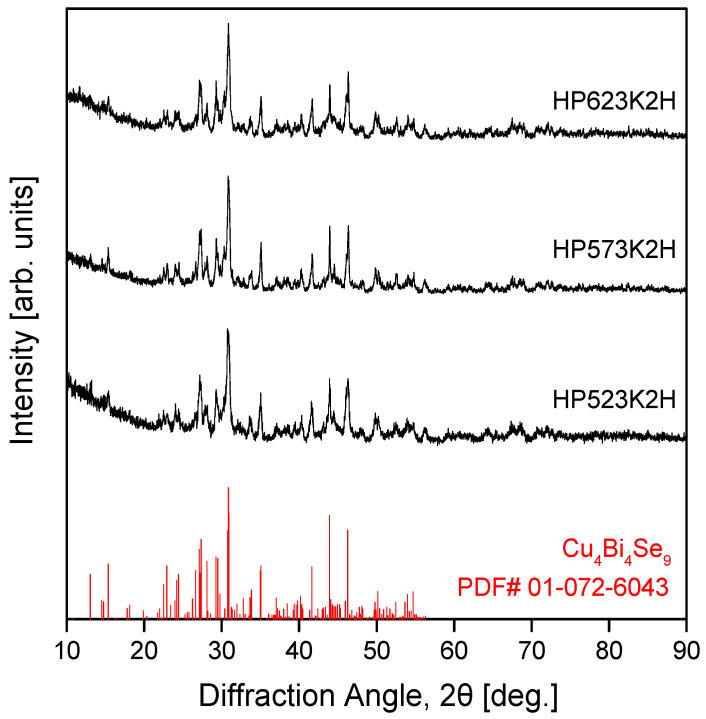
XRD patterns of Cu_4_Bi_4_Se_9_ bulks hot-pressed at 523–623 K for 2 h after 6 h of mechanical alloying. All specimens maintained a single orthorhombic Cu_4_Bi_4_Se_9_ phase without detectable secondary phases.

Figure 2 shows the XRD patterns of samples sintered by HP at 523, 573, and 623 K for 2 h using Cu_4_Bi_4_Se_9_ powders synthesized under the MA 6 h condition (denoted as MA350R6H). All samples exhibited diffraction peaks corresponding to single-phase orthorhombic Cu_4_Bi_4_Se_9_ (PDF# 01-072-6043). The phase identification was confirmed based on full-pattern Rietveld refinement, with no detectable impurity or secondary phases, and identical diffraction features were preserved over the entire sintering temperature range of 523–623 K. These results indicate that the Cu_4_Bi_4_Se_9_ phase formed during MA remains stable against decomposition or phase separation during subsequent HP sintering within this temperature window. With increasing sintering temperature, a slight decrease in peak width accompanied by an increase in peak intensity was observed, reflecting enhanced atomic diffusion during HP that promotes grain growth and partial relaxation of residual microstrain introduced by MA. The absence of peak splitting or additional reflections further suggests that this structural evolution occurs without changes in crystal symmetry or stoichiometry. This behavior is consistent with previous reports in which Cu_4_Bi_4_Se_9_ prepared by the multistep solid-state reaction (SSR) followed by spark plasma sintering (SPS) [8], conventional SSR followed by HP [10], or high-energy ball milling (HEBM) with post-annealing [11] retained a single Pnma phase. Compared with these time- and energy-intensive approaches, the present MA–HP process achieved comparable phase purity and structural stability at lower temperatures and within a shorter processing time. Overall, these results demonstrate that the MA–HP route enables the rapid consolidation of stoichiometric, single-phase Cu_4_Bi_4_Se_9_ with orthorhombic Pnma symmetry without post-annealing, highlighting its effectiveness for low-temperature bulk synthesis.

Table 1 summarizes the lattice constants, relative density, and crystallite size of the Cu_4_Bi_4_Se_9_ samples as a function of HP temperature. All samples retained the orthorhombic structure over the HP temperature range of 523–623 K. With increasing HP temperature, the lattice constants exhibited a slight contraction. Given that all samples were prepared under identical MA conditions, this behavior may not be explained solely by residual stress introduced during MA. It is therefore likely that multiple factors, such as microstrain relaxation, defect rearrangement, and thermally activated atomic diffusion during HP, contribute to the observed lattice contraction. This subtle contraction agrees with the progressive sharpening of diffraction peaks in Figure 2 and reflects improved local structural coherence rather than a change in crystal symmetry. The lattice parameters obtained in this study (a = 3.1102–3.1124 nm, b = 0.3999–0.4006 nm, and c = 1.1392–1.1404 nm) were smaller than those reported for single-crystal Cu_4_Bi_4_Se_9_ by Makovický et al. [7] and were closer to values reported for polycrystalline samples prepared by non-equilibrium routes [8], suggesting that ball-milling-induced lattice strain and fine-grained microstructures result in a slightly contracted average lattice relative to equilibrium single crystals. In contrast to the minor variation in lattice parameters, the relative density decreased markedly from 97.6 to 89.2% with increasing HP temperature, indicating reduced densification efficiency at elevated temperatures, likely due to partial Se volatilization and the associated formation and coarsening of pores. Consistent with this trend, the crystallite size initially increased at lower sintering temperatures as diffusion-assisted grain growth became active, but decreased at higher temperatures as pore formation and defect reaccumulation hindered grain coalescence. This non-monotonic behavior reflects the competition between thermally activated grain growth and volatilization-driven microstructural degradation, with the latter becoming increasingly dominant at higher HP temperatures.

**Figure 3:**
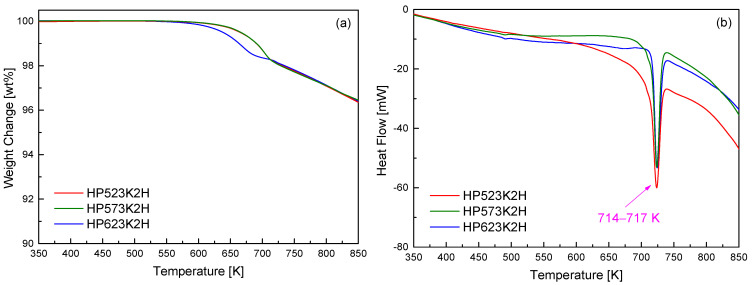
(**a**) TG and (**b**) DSC curves of Cu_4_Bi_4_Se_9_ bulks prepared by MA–HP. Slight mass loss began near 650 K, attributed to partial Se volatilization, while a distinct endothermic peak at 714–717 K marked the onset of the structural destabilization of Cu_4_Bi_4_Se_9_.

Figure 3 presents the TG–DSC results of the Cu_4_Bi_4_Se_9_ samples prepared by the MA–HP process. As shown in the TG curves in Figure 3a, all samples exhibited gradual mass loss beginning at approximately 600 K and continuing up to 850 K, indicating progressive thermal instability associated with volatile species at elevated temperatures. Among them, the HP623K2H specimen showed a slightly earlier onset of mass loss, which correlates with its lower relative density and increased pore development, as summarized in Table 1. Partial Se volatilization during HP at 623 K is therefore considered to contribute to the formation of interconnected micropores and defect networks that act as preferential diffusion pathways, thereby accelerating mass loss during subsequent heating. In contrast, the HP523K2H and HP573K2H samples retained higher densities and more compact microstructures, resulting in delayed and more gradual mass changes at elevated temperatures. Notably, all samples exhibited comparable total mass loss at high temperatures, suggesting that the observed differences mainly arose from surface- or grain-boundary-related processes rather than bulk phase decomposition. The DSC curves in Figure 3b showed a distinct endothermic peak in the narrow temperature range of 714–717 K for all samples, which was attributed to the onset of structural destabilization involving partial decomposition and/or a solid-state phase transition in Cu_4_Bi_4_Se_9_. The sharp and reproducible nature of this feature indicates an intrinsic thermal limit of the Cu_4_Bi_4_Se_9_ framework rather than a processing-induced artifact. The observed transition temperature agrees well with the results reported by Jiang et al. [8], who identified the initiation of structural decomposition near 720 K in Cu_4_Bi_4_Se_9_ prepared by the SSR–SPS. Overall, these findings confirm that the HP temperature range employed in this study (523–623 K) is well below the intrinsic decomposition threshold, providing a reliable processing window in which densification and microstructural evolution can be controlled without compromising the structural integrity of Cu_4_Bi_4_Se_9_.

**Figure 4:**
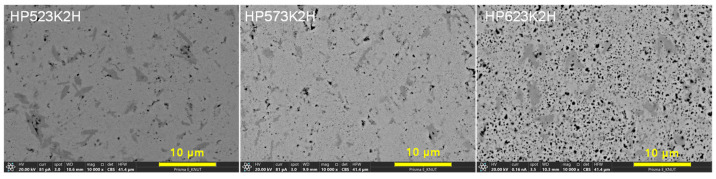
SEM micrographs of the Cu_4_Bi_4_Se_9_ bulks hot-pressed at 523, 573, and 623 K. HP523K2H and HP573K2H exhibited dense and homogeneous microstructures, whereas HP623K2H showed irregular interconnected pores due to Se volatilization and the formation of voids.

Figure 4 presents the SEM micrographs of the Cu_4_Bi_4_Se_9_ specimens sintered at different HP temperatures. The HP523K2H sample exhibited a dense and relatively homogeneous microstructure composed of closely packed submicron grains with minimal residual porosity, consistent with its high relative density. In the HP573K2H specimen, moderate grain growth and improved interparticle bonding were observed, indicating enhanced diffusion during sintering; however, the presence of isolated micropores along certain grain boundaries led to a slight decrease in density. In contrast, the HP623K2H specimen showed pronounced pore development, including irregular and partially interconnected voids distributed throughout the microstructure. This behavior is likely related to Se volatilization at elevated temperature and the associated imbalance in Cu–Se interdiffusion, which may promote the formation of Kirkendall-type voids under non-equilibrium conditions. Similar pore formation associated with chalcogen volatilization has been reported in Cu–Se-based compounds subjected to high-temperature processing [20]. Compared with previous studies in which dense and homogeneous microstructures were achieved through prolonged SSR followed by SPS or HP [8,10], the present MA–HP process yielded comparable microstructural uniformity at 523–573 K, whereas noticeable densification degradation occurred at 623 K. Although a slight brightness contrast was observed in all specimens, it is most reasonably attributed to surface topography or local variations in electron scattering rather than secondary phase formation, as confirmed by the single-phase XRD results in Figure 2. These observations indicate that the HP temperature governs the balance among grain growth, densification, and pore evolution, which in turn influences the transport properties.

**Figure 5:**
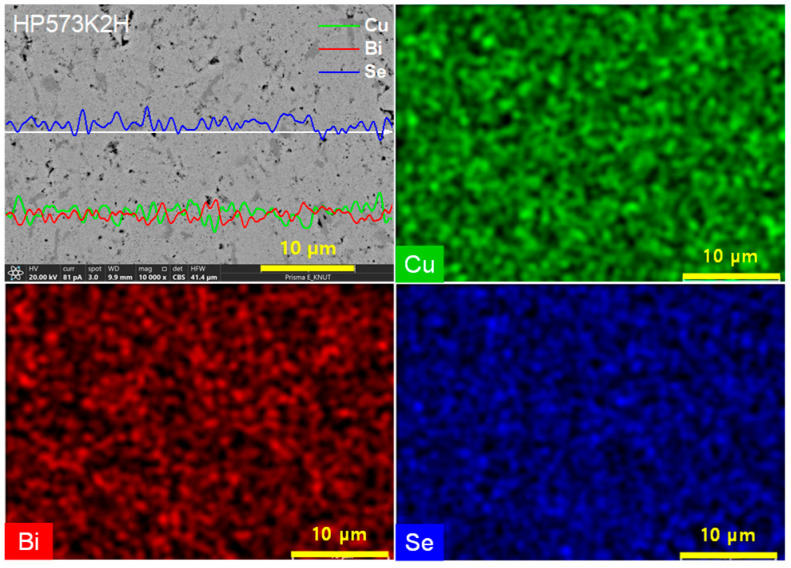
EDS elemental mapping and line-scan analysis of the HP573K2H sample. Cu, Bi, and Se elements were uniformly distributed across the matrix, and no significant compositional fluctuations or secondary-phase segregation were observed.

Figure 5 shows the EDS elemental mapping and line scan results of the HP573K2H sample. Because minor brightness contrast was observed in localized regions in Figure 4, EDS analyses were conducted to evaluate compositional homogeneity and to determine whether the contrast originated from chemical segregation. The elemental maps indicate that Cu, Bi, and Se were uniformly distributed across the examined area, without obvious enrichment or depletion within the spatial resolution of EDS. Line scan profiles across regions with local brightness contrast showed no significant fluctuations in Cu, Bi, or Se signals, suggesting that the contrast is not associated with large-scale compositional segregation. Instead, the observed contrast is more reasonably attributed to microstructural factors such as surface topography, local porosity, or grain orientation, which commonly influence backscattered electron contrast in polycrystalline chalcogenides. These observations suggest that the MA–HP process yields a macroscopically homogeneous elemental distribution. However, due to the limited spatial resolution of EDS, minor compositional variations or secondary phases below the detection limit cannot be excluded. Therefore, the EDS results support overall compositional uniformity, while phase purity was primarily confirmed by XRD. This homogeneous elemental distribution is consistent with previous reports: Jiang et al. [8] observed uniform Cu, Bi, and Se distributions in Cu_4_Bi_4_Se_9_ without impurity phases, while Hobbis et al. [10] similarly confirmed compositional homogeneity and single-phase formation by SEM-EDS analysis. In addition, Han et al. [11] reported homogeneous elemental distributions with an atomic ratio of Cu:Bi:Se ≈ 4:4:9, consistent with stoichiometric single-phase formation. These results indicate that the present MA–HP route produces chemically homogeneous Cu_4_Bi_4_Se_9_, and that the variations in transport properties are governed primarily by microstructural factors rather than compositional fluctuations.

**Figure 6:**
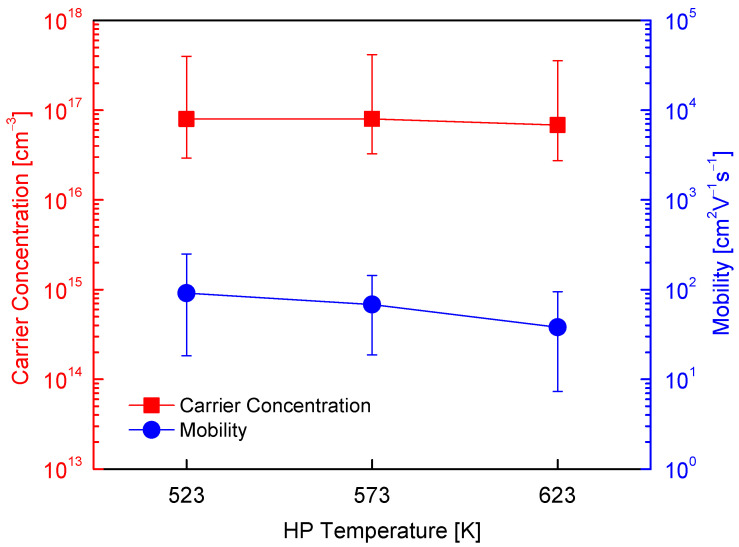
Hall carrier concentration (n) and mobility (μ) of Cu_4_Bi_4_Se_9_ as a function of hot-pressing temperature. All samples exhibited p-type conduction with n ≈ 10^17^ cm^−3^ and μ ≈ 10^2^ cm^2^ V^−1^ s^−1^.

Figure 6 shows the Hall carrier concentration and mobility of Cu_4_Bi_4_Se_9_ as a function of HP temperature. All samples exhibited intrinsic p-type conduction, with carrier concentrations on the order of 10^17^ cm^−3^ and carrier mobilities on the order of 10^2^ cm^2^ V^−1^ s^−1^. As the HP temperature increased from 523 to 623 K, the carrier concentration decreased slightly, whereas the mobility showed a more pronounced and monotonic decline. This contrasting trend suggests that carrier transport in Cu_4_Bi_4_Se_9_ is influenced more strongly by microstructural scattering than by variations in carrier generation. In particular, the lower mobility observed for the HP623K2H sample correlates with its reduced relative density and enhanced pore development, which can disrupt continuous charge transport pathways and increase carrier scattering at pore surfaces and grain boundaries, as shown in Table 1 and Figure 4. In contrast, the weak dependence of carrier concentration on HP temperature reflects the intrinsic defect chemistry of Cu_4_Bi_4_Se_9_, where Cu vacancies ($V_{Cu}^{\prime }$) within the Cu–Se network act as the primary source of holes [8], and trivalent cation antisite defects at Cu sites, such as ${Sb}_{Cu}^{\cdot \cdot }$ or ${Bi}_{Cu}^{\cdot \cdot }$ in related Cu–Sb and Cu–Bi chalcogenides, may also function as acceptors [9]. The absence of significant compositional variation or secondary phases, as confirmed by XRD and EDS analyses, suggests that the concentration of these intrinsic acceptor-type defects remains largely unchanged over the HP temperature range of 523–623 K. For comparison, Jiang et al. [8] reported a similar carrier concentration (8.4 × 10^16^ cm^−3^) but a substantially higher mobility (5.36 × 10^3^ cm^2^ V^−1^ s^−1^) for Cu_4_Bi_4_Se_9_, which they attributed to near-single-crystal-like crystallinity and a microstructure favorable for long-range charge transport. This comparison indicates that in polycrystalline Cu_4_Bi_4_Se_9_ prepared by rapid consolidation routes, mobility (consequently electrical transport) is primarily limited by microstructural factors rather than intrinsic carrier density.

**Figure 7:**
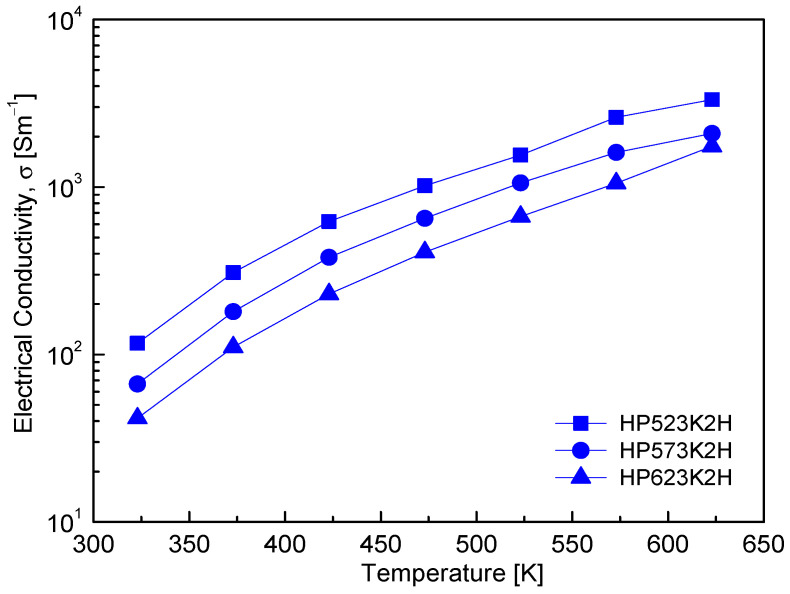
Temperature-dependent electrical conductivity (σ) of Cu_4_Bi_4_Se_9_. σ increases monotonically with temperature, indicating typical non-degenerate semiconducting behavior.

Figure 7 shows the temperature dependence of the electrical conductivity of Cu_4_Bi_4_Se_9_. For all samples, the electrical conductivity increased monotonically with temperature, rising from approximately 10^2^ S m^−1^ at 323 K to about 10^3^ S m^−1^ at 623 K, which is characteristic of thermally activated transport in non-degenerate p-type semiconductors. This trend agrees with the results reported by Jiang et al. [8], who observed an increase from 1.95 × 10^2^ S m^−1^ at 300 K to 7.33 × 10^2^ S m^−1^ at 530 K. The temperature dependence reflects thermally activated holes associated with intrinsic acceptor-type defects, such as Cu vacancies and antisite defects, within the Cu–Se bonding network, consistent with the intrinsic p-type semiconducting behavior of Cu_4_Bi_4_Se_9_ [11]. In contrast, variations in electrical conductivity with respect to HP temperature arise mainly from microstructural effects rather than changes in carrier generation. As summarized in Table 1 and shown in Figure 4, increasing the HP temperature leads to reduced relative density, pore development, and weakened grain boundary connectivity, which can disrupt percolative charge transport pathways in the polycrystalline bulk. Although partial Se volatilization at elevated HP temperatures could potentially alter the hole concentration, Hall measurements (Figure 6) indicate that the carrier concentration remained nearly constant at ~10^17^ cm^−3^ over the HP temperature range. This suggests that the decrease in electrical conductivity with increasing HP temperature is primarily associated with reduced carrier mobility rather than variations in carrier density. Accordingly, enhanced carrier scattering at the pores, grain boundaries, and defect clusters introduced or intensified during high-temperature sintering appears to be the dominant factor governing the HP-temperature-dependent electrical transport behavior in Cu_4_Bi_4_Se_9_.

**Figure 8:**
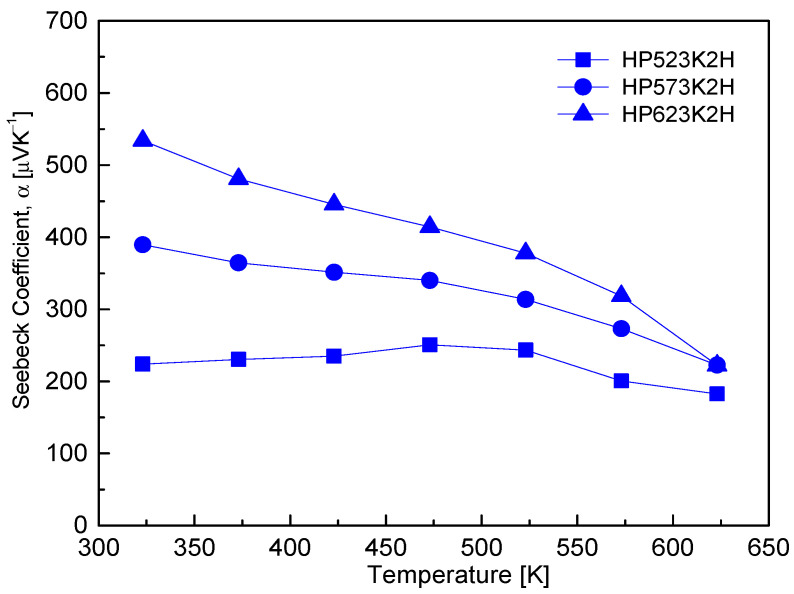
Temperature dependence of the Seebeck coefficient (α) for Cu_4_Bi_4_Se_9_. All samples showed positive α values, confirming p-type conduction.

Figure 8 shows the temperature dependence of the Seebeck coefficient of Cu_4_Bi_4_Se_9_. All samples exhibited positive Seebeck coefficients over the entire temperature range, confirming intrinsic p-type conduction. The Seebeck coefficient decreased monotonically with increasing temperature, ranging from 223–534 μV K^−1^ at 323 K and converging to 182–222 μV K^−1^ at 623 K, which is characteristic of non-degenerate semiconductors where thermally activated carriers progressively reduce the energy asymmetry of charge transport. In addition to this temperature dependence, a clear dependence on HP temperature was observed, with samples sintered at higher HP temperatures showing relatively larger Seebeck coefficients. This trend is consistent with the carrier concentration data in Figure 6 and the electrical conductivity results in Figure 7, in accordance with the Pisarenko relation, whereby a lower hole concentration corresponds to a higher Seebeck coefficient [21]. The relatively weak temperature dependence of the Seebeck coefficient, compared with the strong increase in electrical conductivity, can be understood from their different dependence on carrier concentration. The electrical conductivity follows σ = n·e·μ, indicating a linear dependence on carrier concentration, whereas the Seebeck coefficient can be expressed as α = (8/3)π^2^·k_B_^2^·e^−1^·h^−2^·m*·T·(π/3n)^2/3^, showing an inverse dependence on n^2/3^, where k_B_, h, and m* are the Boltzmann constant, the Planck constant, and the effective carrier mass, respectively. Using the Pisarenko relation, the effective carrier mass was estimated from the experimentally obtained Seebeck coefficient and Hall carrier concentration at room temperature. The calculated m*/m_o_ values ranged from approximately 0.02 to 0.04, indicating an unusually small effective mass. This suggests that the valence band of Cu_4_Bi_4_Se_9_ is highly dispersive, which is generally favorable for achieving high carrier mobility. Despite this advantageous band structure, the electrical conductivity remained relatively low due to the limited carrier concentration (~10^17^ cm^−3^), implying that carrier density rather than band structure is the primary factor governing the thermoelectric transport properties in this system.

Therefore, even when the carrier concentration exhibits only weak temperature dependence, α remains relatively insensitive to temperature, while σ can increase significantly due to thermally activated transport. Although partial Se volatilization at elevated HP temperatures could introduce local compositional variations and partial hole compensation, Hall measurements indicate that the overall change in carrier concentration is limited, suggesting that variations in energy-dependent carrier scattering and transport pathways also contribute to the observed differences in Seebeck coefficient. The gradual decrease in the Seebeck coefficient with increasing temperature can be attributed to the shift in the Fermi level toward the conduction band due to thermal carrier excitation, together with possible changes in the dominant scattering mechanism at elevated temperatures. For comparison, Hobbis et al. [10] reported a Seebeck coefficient of approximately 1 mV K^−1^ at room temperature for Cu_4_Bi_4_Se_9_, while Jiang et al. [8] observed typical p-type behavior with Seebeck coefficients of 500–600 μV K^−1^ between 300 and 550 K, followed by a gradual decrease at higher temperatures, which they attributed to thermal carrier excitation and the onset of intrinsic conduction associated with increased interband transport.

**Figure 9:**
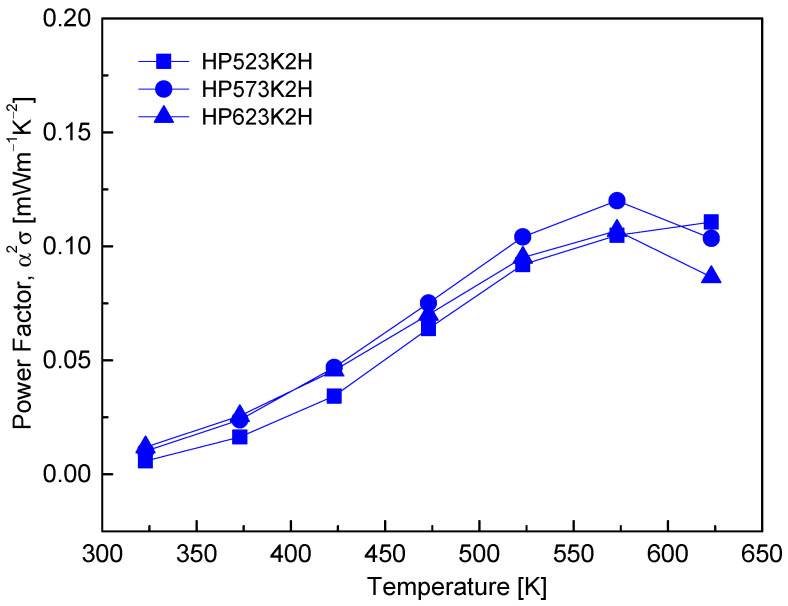
Power factor (α^2^·σ) of Cu_4_Bi_4_Se_9_ as a function of temperature. The power factor increased with temperature and peaked around 573–623 K, achieving a maximum for the HP573K2H sample, governed by the interplay between σ and α.

Figure 9 shows the temperature dependence of the power factor of Cu_4_Bi_4_Se_9_. For all samples, the power factor increased with temperature and reached a maximum in the range of 573–623 K, followed by slight saturation or a small decrease at higher temperatures for some specimens. Among them, the HP573K2H sample exhibited the highest power factor, attaining a maximum value of 0.12 mW m^−1^ K^−2^ at 573 K, indicating a favorable balance between electrical conductivity and the Seebeck coefficient under this sintering condition. This temperature-dependent behavior reflects the competing contributions of electrical conductivity and thermopower (Seebeck coefficient): at lower temperatures, the thermally activated increase in electrical conductivity (Figure 7) predominates, leading to continuous enhancement of the power factor, whereas at higher temperatures, the more rapid decrease in the Seebeck coefficient (Figure 8) gradually offsets further gains in conductivity. The slight suppression of the power factor at elevated temperatures for samples sintered at higher HP temperatures can be associated with microstructural degradation, including increased porosity and reduced carrier mobility, which limit the effective contribution of thermally activated carriers. Compared with the HP523K2H and HP623K2H samples, the HP573K2H specimen achieved a more favorable compromise between densification and defect-related scattering, resulting in the highest power factor. For comparison, Jiang et al. [8] reported a continuously increasing power factor reaching 0.137 mW m^−1^ K^−2^ at 530 K for Cu_4_Bi_4_Se_9_ synthesized by prolonged SSR followed by SPS, a value comparable to that obtained in the present study. These results suggest that despite the shorter processing time and lower synthesis temperature, the MA–HP route can produce Cu_4_Bi_4_Se_9_ with competitive power factor performance, comparable to that of related ternary chalcogenides in the (Cu/Ag)–(Bi/Sb)–(S/Se) system.

**Figure 10:**
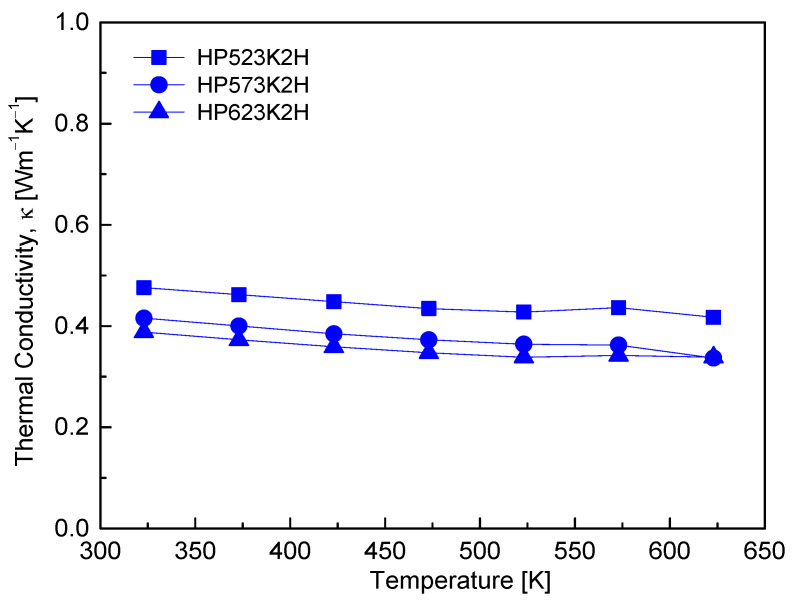
Temperature-dependent thermal conductivity (κ) of Cu_4_Bi_4_Se_9_. κ decreased slightly with temperature, maintaining extremely low values throughout the measured range.

Figure 10 shows the temperature dependence of the thermal conductivity of Cu_4_Bi_4_Se_9_. Over the temperature range of 323–623 K, all samples exhibited a gradual decrease in thermal conductivity with increasing temperature, maintaining low values in the range of 0.33–0.48 W m^−1^ K^−1^. This behavior is characteristic of phonon-dominated heat transport, where enhanced phonon–phonon (Umklapp) scattering at elevated temperatures reduces the phonon mean free path. In addition to this intrinsic temperature effect, the thermal conductivity decreases systematically with increasing HP temperature, indicating a significant influence of microstructural evolution on heat transport. As summarized in Table 1 and shown in Figure 4, higher HP temperatures result in reduced relative density, increased microporosity, and a greater density of structural defects, which serve as effective phonon scattering centers. In particular, the lower thermal conductivity observed for the HP623K2H sample correlates with pore formation and density reduction, suggesting that the disruption of continuous heat transport pathways by pores contributes to the suppression of phonon propagation. Beyond these extrinsic microstructural effects, Cu_4_Bi_4_Se_9_ intrinsically exhibits low lattice thermal conductivity due to its complex orthorhombic Pnma crystal structure, which involves strong anharmonic lattice vibrations associated with Bi 6s^2^ lone-pair electrons, as well as dynamic disorder and localized low-frequency vibrations (rattling-like motion) originating from partially occupied Cu sites. The combined influence of intrinsic anharmonicity and microstructure-induced phonon scattering accounts for the low thermal conductivity observed in all samples, providing a favorable basis for thermoelectric performance in this system.

**Figure 11:**
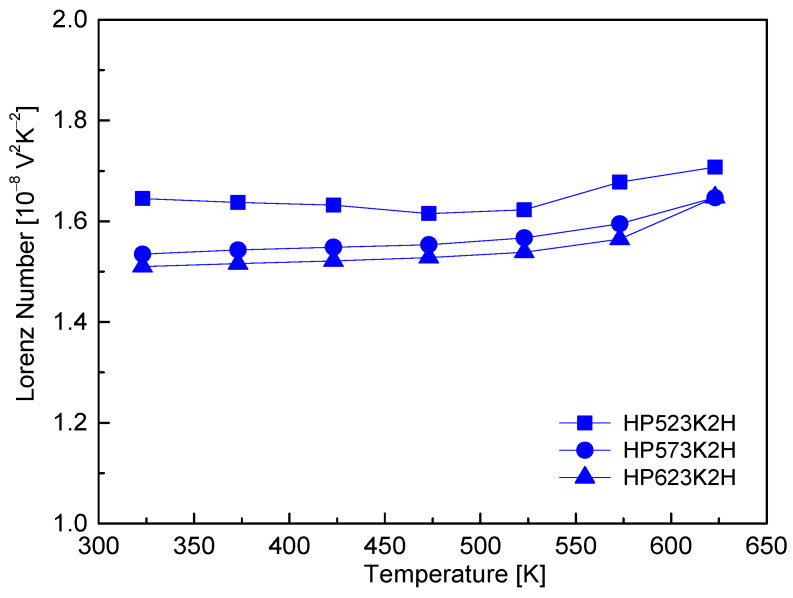
Lorenz number (L) of Cu_4_Bi_4_Se_9_ calculated using the single parabolic band model. L values lie well below the metallic Wiedemann–Franz limit, confirming non-degenerate semiconducting transport.

Figure 11 shows the Lorenz number (L) of Cu_4_Bi_4_Se_9_, calculated from the relationship ($L=1.5+exp(-\left|\alpha \right|/116$) between the Lorenz number and the Seebeck coefficient based on the single parabolic band model [22]. Over the temperature range of 323–623 K, L increased gradually with temperature, taking values from 1.51 to 1.70 × 10^−8^ V^2^ K^−2^. These values were substantially lower than the Wiedemann–Franz limit for metals (L ≈ 2.44 × 10^−8^ V^2^ K^−2^), clearly indicating that charge transport in Cu_4_Bi_4_Se_9_ occurs in a non-degenerate semiconducting regime rather than a metallic or heavily degenerate state. The gradual increase in L with temperature reflects the progressive thermal excitation of carriers and the associated evolution of scattering mechanisms, which is consistent with the observed temperature dependence of the Seebeck coefficient and electrical conductivity. Using these Lorenz numbers, the electronic thermal conductivity (κ_E_ = L·σ·T) was evaluated, and the lattice thermal conductivity (κ_L_) was obtained by subtracting κ_E_ from the total thermal conductivity (κ). The calculated κ_E_ accounted for less than 10% of κ over the entire temperature range, resulting in nearly identical values of κ and κ_L_. Therefore, for clarity, only κ is presented in Figure 10. This confirms that heat transport in Cu_4_Bi_4_Se_9_ is predominantly governed by lattice (phonon) conduction, and that the observed ultralow thermal conductivity originates mainly from phonon-related scattering rather than electronic heat transport. Consistent with this conclusion, Jiang et al. [8] reported a very low lattice thermal conductivity (κ_L_) of 0.29–0.35 W m^−1^ K^−1^ for Cu_4_Bi_4_Se_9_ between 300 and 500 K, which they attributed to the combined effects of a complex crystal structure, weak Cu–Se bonding, Cu-atom rattling, and strong anharmonicity associated with Bi 6s^2^ lone-pair electrons. Similarly, Hobbis et al. [10] observed a nearly temperature-independent low thermal conductivity of approximately 0.38–0.40 W m^−1^ K^−1^ in the range of 300–600 K and emphasized that the high electrical resistivity resulted in a negligible electronic contribution. The intrinsically low lattice thermal conductivity of Cu_4_Bi_4_Se_9_, rather than electronic heat transport, is the dominant factor governing its thermal behavior and plays a central role in enabling enhanced thermoelectric performance.

**Figure 12:**
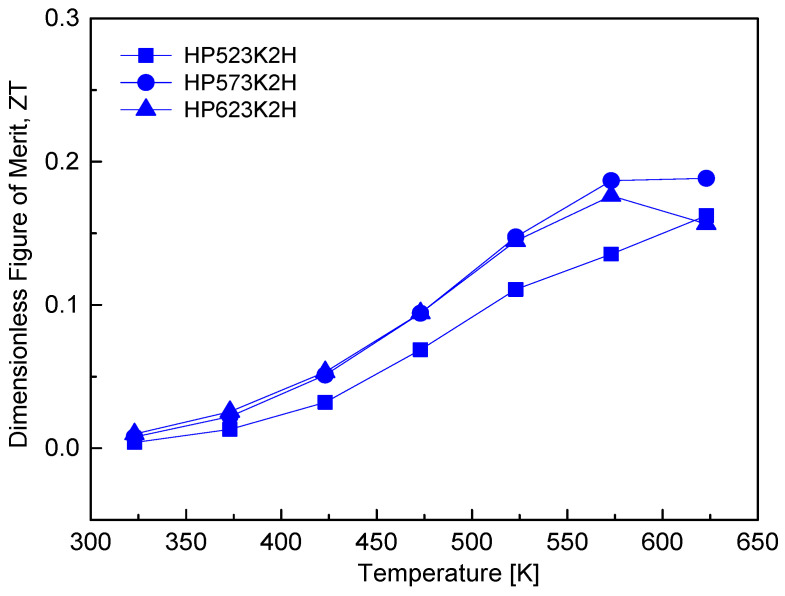
Temperature dependence of the dimensionless thermoelectric figure of merit (ZT) for Cu_4_Bi_4_Se_9_. ZT increased with temperature and reached a maximum at 573–623 K. The superior performance arises from the balance between ultralow κ and moderate power factor achieved via the MA–HP process.

Figure 12 shows the temperature dependence of the dimensionless thermoelectric figure of merit (ZT) for Cu_4_Bi_4_Se_9_. For all samples, ZT increased monotonically with temperature, with the HP573K2H specimen reaching a maximum ZT of 0.19 in the range of 573–623 K. This trend reflects the combined effects of the temperature-dependent power factor and thermal conductivity, which evolve in opposite directions as temperature increases. The rise in ZT at elevated temperatures is mainly associated with the strong suppression of lattice thermal conductivity, whereas the improvement in the power factor becomes limited due to the concurrent reduction in carrier mobility and Seebeck coefficient. The dependence of ZT on HP temperature further illustrates this trade-off: although higher HP temperatures reduce thermal conductivity through increased porosity and defect-induced phonon scattering, they also deteriorate the power factor by disrupting carrier transport pathways, thereby limiting further enhancement of ZT. Consequently, the HP573K2H sample achieved the most balanced combination of electrical and thermal transport properties, resulting in the highest ZT among the specimens studied. For comparison, Jiang et al. [8] reported a higher peak ZT of 0.24 at 530 K for Cu_4_Bi_4_Se_9_ prepared by the prolonged multistep solid-state reaction followed by spark plasma sintering (SSR–SPS), which required approximately 8 days, and attributed the improved performance to ultralow lattice thermal conductivity and optimized electrical transport. Their single parabolic band model calculations further suggest that increasing the carrier concentration to approximately 10^21^ cm^−3^ could raise the ZT to about 0.8 at 511 K, highlighting the strong sensitivity of ZT in Cu_4_Bi_4_Se_9_ to carrier concentration. In the present study, the comparatively lower ZT values were associated with the lower carrier concentration, which limits the attainable power factor despite the low lattice thermal conductivity. Nevertheless, achieving a ZT of 0.19 using the MA–HP process demonstrates that competitive thermoelectric performance can be obtained through a simple and rapid synthesis route without post-annealing, underscoring the practical potential of MA–HP processing for scalable fabrication of Cu_4_Bi_4_Se_9_-based thermoelectric materials.

## 4. Conclusions

Bulk Cu_4_Bi_4_Se_9_ specimens were synthesized by combining mechanical alloying and hot pressing, and their phase formation behavior, microstructural evolution, charge transport characteristics, and thermoelectric properties were systematically examined. X-ray diffraction analysis confirmed that all powders milled for 3–12 h crystallized into a single-phase orthorhombic Pnma structure without post-annealing, and this phase stability was maintained after hot pressing, with no evidence of phase decomposition or secondary phase formation. Thermal stability analysis revealed a distinct endothermic peak at 714–717 K, corresponding to the onset of the structural destabilization of Cu_4_Bi_4_Se_9_, indicating that the hot-pressing temperature range employed in this study lies below the decomposition threshold. As the hot-pressing temperature increased from 523 to 623 K, grain growth accompanied by pore formation led to a decrease in relative density from 97.6% to 89.2%. All samples exhibited intrinsic p-type conduction, with carrier concentrations on the order of 10^17^ cm^−3^ and carrier mobilities of approximately 10^2^ cm^2^ V^−1^ s^−1^. The electrical conductivity increased monotonically with temperature, consistent with non-degenerate semiconducting behavior, whereas the Seebeck coefficient gradually decreased, resulting in a maximum power factor of 0.12 mW m^−1^ K^−2^ at 573 K. The thermal conductivity remained low, in the range of 0.33–0.48 W m^−1^ K^−1^, with lattice thermal conductivity as the dominant contribution. The low lattice thermal conductivity is likely associated with enhanced anharmonic phonon scattering, which in Cu–Bi chalcogenides is generally linked to intrinsic structural features such as Cu-atom rattling, Bi 6s^2^ lone-pair electrons, and the complexity of the orthorhombic lattice. Consequently, Cu_4_Bi_4_Se_9_ achieved a maximum dimensionless figure of merit ZT of 0.19 in the temperature range of 573–623 K. These results indicate that a stable single phase and competitive thermoelectric performance can be achieved through a simple and rapid MA–HP process without post-annealing, demonstrating its potential as a practical synthesis route for bulk Cu_4_Bi_4_Se_9_ thermoelectric materials.

## Figures and Tables

**Table 1:** Relative densities, crystallite sizes, and lattice parameters of Cu_4_Bi_4_Se_9_ samples hot-pressed at different temperatures. All samples crystallized in the orthorhombic structure.

Specimen	Relative Density [%]	Crystallite Size [nm]	Lattice Constant [nm]		
			a	b	c
HP523K2H	97.6	19.9	3.1124(2)	0.4006(7)	1.1404(1)
HP573K2H	96.8	26.0	3.1119(1)	0.4004(4)	1.1394(8)
HP623K2H	89.2	21.5	3.1102(2)	0.3999(5)	1.1392(1)

## Data Availability

The original contributions presented in this study are included in the article; further inquiries can be directed to the corresponding author.
